# Enriched environment exposure during development positively impacts the structure and function of the visual cortex in mice

**DOI:** 10.1038/s41598-023-33951-0

**Published:** 2023-04-29

**Authors:** O. Bibollet-Bahena, S. Tissier, S. Ho-Tran, A. Rojewski, C. Casanova

**Affiliations:** Laboratoire des Neurosciences de la Vision, School of Optometry, Université de Montréal, Montreal, QC Canada

**Keywords:** Neuroscience, Development of the nervous system, Visual system

## Abstract

Optimal conditions of development have been of interest for decades, since genetics alone cannot fully explain how an individual matures. In the present study, we used optical brain imaging to investigate whether a relatively simple enrichment can positively influence the development of the visual cortex of mice. The enrichment paradigm was composed of larger cages housing multiple mice that contained several toys, hiding places, nesting material and a spinning wheel that were moved or replaced at regular intervals. We compared C57BL/6N adult mice (> P60) that had been raised either in an enriched environment (EE; n = 16) or a standard (ST; n = 12) environment from 1 week before birth to adulthood, encompassing all cortical developmental stages. Here, we report significant beneficial changes on the structure and function of the visual cortex following environmental enrichment throughout the lifespan. More specifically, retinotopic mapping through intrinsic signal optical imaging revealed that the size of the primary visual cortex was greater in mice reared in an EE compared to controls. In addition, the visual field coverage of EE mice was wider. Finally, the organization of the cortical representation of the visual field (as determined by cortical magnification) versus its eccentricity also differed between the two groups. We did not observe any significant differences between females and males within each group. Taken together, these data demonstrate specific benefits of an EE throughout development on the visual cortex, which suggests adaptation to their environmental realities.

## Introduction

One of the main functions of the brain is to process information from the environment. To reach that goal, a complex network of connections self-assembles between billions of neurons through a finely orchestrated series of stages. Brain development is regimented by its genetic blueprint and through interactions with the environment. There is an impressive level of plasticity that allows for optimal development. Since Rosenzweig’s studies in the 1960s^1–3^, human and animal studies alike have revealed important environmental factors involved and their effects. Relative enrichment of an environment compared to another (housing conditions) has been a central paradigm for animal research on experience-dependent development^1,4,5^. Enrichment is based on complexity and novelty with inanimate objects (toys, hiding places and spinning wheels, for instance) being moved or replaced at regular intervals, as well as on social interactions. It allows for enhanced stimulation of sensory, motor and cognitive modalities. Rodent studies have shown that brain size and weight^2,6,7^, as well as the cortical thickness^8^, increase with environmental enrichment. At the molecular level, an enriched environment (EE) induces the expression of growth factors such as neurotrophins (known to be implicated in young and adult plasticity^9–12^) and insulin-like growth factor-1 (IGF-1; involved in mediating growth and development^13,14^). At a higher scale, an EE causes an increase in dendritic arborization^15^, and synapse size and number in various cortical areas^16–20^. Studies have also demonstrated that exposure to an EE promotes neuronal plasticity^21^ and functional recovery^22^. Furthermore, maternal care towards the pups is improved in an EE^23^.

To date, little is known about the developmental effects of an EE on the functional structure of the visual cortex. Previous studies have demonstrated that an EE accelerates the development of the visual brain of rodents. More specifically, when placed in an EE, visual acuity of mice develops 6 days in advance—an effect mediated through BDNF, IGF-1 and the GABAergic system^14,23–25^ and is ultimately higher in average^26^ compared with standard reared mice. Ocular dominance plasticity is also prolonged into adulthood of mice raised in an EE^27^. Wild-caught rats, which could be considered an extreme level of EE, exhibit higher densities of neurons in the visual cortex compared to laboratory rats^28^. In addition, Bartoletti and colleagues (2004) have shown that the effects of dark rearing on the rat visual cortex are prevented by an EE, which allows for proper consolidation of visual cortical connections^29^. Moreover, environmental impoverishment, such as a reduced sensory-motor stimulation during development, profoundly affects visual acuity and visual evoked potential latency development compared to standard reared mice^30^. Cang and colleagues also demonstrated that monocular visual deprivation during development induces ocular dominance plasticity^31^.

These studies have highlighted the exceptional level of plasticity during development^32^. All developmental stages have critical periods identified relating to an EE, namely prenatal (maternal experience during gestation), early postnatal (pre-weaning) and late postnatal (post-weaning) (reviewed in^32^). Although it is possible to reopen windows of plasticity in the adult mouse, as shown by periods of exercise prior to recordings assessing ocular dominance^33^, these effects are not as drastic as during development. Previous studies in humans and animal models have emphasized that an EE accelerates aspects of development, but also increases the length of the window of plasticity, affecting the pace of brain development (reviewed in^34^). Moreover, data suggests that heritability has a spatiotemporal component with phylogenetically older areas developing first and being progressively less affected by genetics as adulthood is reached (for instance, the primary sensory cortex compared to the association cortex in humans^35^). Genetic predispositions of brain development have been studied in monozygotic twin pairs. Analyses have often focused on total brain or region-specific volumes. However, measures of volume are influenced by both cortical thickness and surface area, which are genetically uncorrelated^36^. Recent findings show that prenatal/perinatal periods are sensitive periods for cortical surface area development^37^. Nonetheless, more work needs to be done in order to have a better understanding of brain development as, overall, surface area is often overlooked, and more so functional delimitations (reviewed in^34^).

An effective and non-invasive method of evaluating cortical visual functions is intrinsic signal optical imaging (ISOI)^38^. Retinotopic mapping is readily obtained through temporally encoded maps of hemodynamic responses to identify the primary visual cortex (V1) and the extrastriate cortex^39–41^. The constant developments in data acquisition and analysis from ISOI^42,43^ and other imaging techniques—functional magnetic resonance^44^ and calcium imaging^45^—have allowed a more thorough examination of the recordings and characterization of the cortical visual system. However, different challenges in the precision of the delimitations of the areas activated through ISOI remain given the low signal-to-noise ratio. The addition of three texture analysis techniques to our pipeline—the entropy, standard deviation and range of the signals—renders clearer border definitions from the activated and non-activated area boundaries, and therefore more robust delimitations.

In the present study, we used optical brain imaging to determine the effects of an EE throughout development on the structure and function of the visual cortex of mice. Our results provide evidence that EE has a positive impact on the functional organization of the primary visual cortex (V1).

## Materials and methods

### Animals

Nine C57BL/6N pregnant mice were obtained from Charles River (Saint-Constant, Qc, Canada) 1 week before the due date. Mice were housed in a controlled environment with a 12 h light/dark cycle with food and water ad libitum. All procedures were carried out in agreement with the guidelines of the Canadian Council for the Protection of Animals, and the experimental protocol was approved by the Ethics Committee of the Université de Montréal. All methods were carried out in accordance with ARRIVE guidelines. Females with litters pertaining to the enriched environment (EE) group were placed in cages accordingly as soon as they were received (two females per cage; 6 females in total), whereas females with litters pertaining to the standard (ST) group were placed individually in smaller cages (3 females in total). ST mice (n = 12) and mice that were exposed to an EE from birth (n = 16) were compared during adulthood (P69-P114). The EE consisted in group housing in larger cages (dimensions: 50 × 38 × 20 cm) containing several toys of different materials (plastic, carton, wood), hiding places, nesting material and a spinning wheel whose positions were changed within the cage and replaced once a week each at regular intervals (changes on Mondays and replacements on Thursdays). Following weaning, ST mice were individually housed in standard cages (dimensions: 30 × 19 × 12.5 cm) with only nesting material (Supplementary Fig.  S1). Mice remained in their respective environments until the day of data acquisition.

### Surgical procedures

Adult animals were first weighted (see Supplementary Table S1 for mouse weights) and sedated with intraperitoneal chlorprothixene (5 mg/kg) to allow administration of a lower dose of the anesthetic. Mice were then anesthetized with intraperitoneal urethane (1 g/kg, in saline) 30 min later. The subsequent surgical procedures were as previously described in Oliveira Ferreira Souza and colleagues^46^. Briefly, atropine (0.05 mg/kg) was administered subcutaneously to reduce tracheal secretion and to counteract the parasympathomimetic effects of the anesthesia. Injectable lidocaine (0.2%) was used at incision sites, whereas lidocaine gel was used at all pressure points. For better animal condition under prolonged anesthesia, a tracheotomy was performed^47^. Animals were then placed on a stereotaxic apparatus, and a constant flow of oxygen was placed in front of the tracheal tube. Viscous artificial tears were frequently applied to avoid corneal dehydration. The scalp and connective tissue were removed to expose the occipital portion of the skull. The mouse cortex was imaged through the skull. A 10 mm wide metal imaging chamber was glued over the skull. Low melting point agarose (1% in saline) was used to fill the chamber, which was then sealed with a glass cover slip. Cardiac activity (by electrocardiogram with subdermal electrodes) and core body temperature (maintained around 37 °C using a heating pad feedback-controlled by a rectal thermoprobe) were monitored throughout the experiment. At the end of the experiments, animals were killed by an overdose of urethane.

### Visual stimuli

Visual stimuli were projected on a flat translucent screen 14 cm from the animal's eyes covering 148° by 113° of the visual field. The mouse eye was aligned with the center of the screen horizontally and placed 10 cm from the bottom of the projection. The luminance of the screen ranged from 0.29 cd/m^2^ (black) to 50 cd/m^2^ (white). Stimuli were generated by the Vpixx software (version 3.20, Vpixx Technologies, Saint-Bruno, QC, Canada). Continuous periodic stimulation to generate retinotopic maps consisted in a vertical or horizontal 20° wide bar spanning the full length of the screen in the orientation of propagation (corrected spherically for projection on a planar screen) drifting over a gray background in four directions (0, 90, 180 and 270°) at 0.15 Hz for 800 s^40,45^. The bar contained a black-and-white checkerboard pattern flickering at 6 Hz between black and white 25° squares to better stimulate the visual system. The stimulations were presented monocularly (screen at a 60° angle from the mouse’s midline). The order of stimulations was randomized.

### Data acquisition and processing

Images were captured using a CMOS camera (Photonfocus A1312, Switzerland) coupled to a macro lens (Nikon, AF Micro Nikkor, 60 mm). Images were sampled at 7.5 Hz (exposure time of the camera of 33 ms with all frames averaged to 7.5 Hz) at a resolution of 1312 × 1082 pixels (spatial resolution of 5.5 µm × 5.5 µm/pixel). Data acquisition was controlled by a Brain Imager 3001 system through the LDAQ software (Optical Imaging Ltd., Rehovot, Israel). Anatomic references were made under illumination at 545 ± 20 nm wavelength. Intrinsic signal recordings were performed under illumination at 630 ± 30 nm wavelength at a focus of approximately 100 µm below the cortical surface. Data analysis was performed through custom scripts in MATLAB (version R2017b, The MathWorks, Inc., Natick, Massachusetts, United States).

Images were coregistered to remove movement artifacts using only rigid transformations. A global signal regression was then performed to remove any light fluctuations. For each direction recorded, we performed a Fourier transform at the frequency of the visual stimulation to extract the phase and amplitude component of the signal^40^. Relative retinotopic maps in opposite directions were corrected for the hemodynamic delay to produce retinotopic maps in each orientation (azimuth and elevation) relative to neural activity. Maps of the sine of the difference between the azimuth and elevation retinotopic gradients were then generated (visual field sign) to identify cortical visual areas^44,48^. We established borders at reversals of the visual field sign at peripheral representations^41^. Following this step, three members of the laboratory independently delineated each map (vertical or horizontal retinotopies) through the analysis of the combination of different maps previously generated (phase, amplitude and hemodynamic delay) and texture analysis techniques (entropy, standard deviation and range) without knowing which group each mouse pertained to. Each pixel was then evaluated. At least two people had to have selected a given pixel for it to be considered within the activated area. The overlap of this considered area had to be over 70% of the total area delimited. Once the consensus delimitations made, we measured the level of overlap between the vertical and the horizontal map for each cortical hemisphere for each mouse. Only data from mice for which there was an overlap greater than 70% were further analyzed to allow for good segmentation of V1 and extrastriate areas. Moreover, data from one hemisphere per mouse was kept (the acquisitions that showed greater overlap, which correlated to greater signal-to-noise ratio). A total of 41 mice were assessed in this study. This amounted to 16 out of 21 mice for the EE group (76%), and 12 out of 20 mice for the ST group (60%). Subsequent analyses were based on the housing environment of the mice (ST or EE); we also made comparisons based on gender within each group. We calculated the cortical area activated (total, V1 and lateral extrastriate areas – namely, the anterolateral area, the laterointermediate area, the lateral anterior area, the lateromedial area and the rostrolateral area). We then determined the following in V1: the amplitude of the signal, the visual field coverage, the range of vision in azimuth and elevation as previously assessed in our laboratory^46^, the scatter index as determined by Cang and colleagues^49^, and the cortical magnification factor (as well as the cortical magnification factor versus the eccentricity and cortical area versus the eccentricity) using similar methodology as Garrett and colleagues^42^, as explained throughout the Results section.

### Statistics

To determine whether data were normally distributed within groups, the Kolmogorov–Smirnov test was performed. When data were normally distributed, comparisons were made using unequal variances *t*-tests (Welch’s t-test). Otherwise, Wilcoxon Rank-sum tests were performed. Our main assumption being that mice reared in an EE had a developmental advantage since brain size and weight is increased with EE, we considered one-tailed comparisons. However, for amplitude and for gender comparisons, we performed two-tailed comparisons, as we found no indication in the literature for the direction of change. *P*-values less than 0.05 were considered significant (corrections were made for multiple testing when appropriate using the Bonferroni correction). JASP statistical package (version 0.14.1.0, JASP Team, Amsterdam, The Netherlands) and Microsoft Excel software (version 16.0, Microsoft Inc., Redmond, WA, USA) were used as complementary statistical tools.

## Results

### Size of visual cortical areas

**Figure 1:**
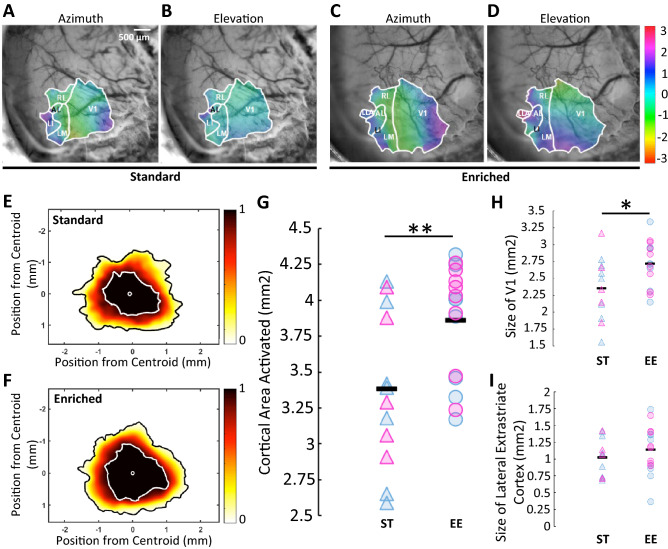
Cortical area activated was determined through retinotopic mapping. Representative maps in azimuth (**A**) and elevation (**B**) of standard mice compared to representative maps in azimuth (**C**) and elevation (**D**) of mice that grew in an enriched environment. Aligned delimitations of the visual cortex (V1 and lateral extrastriate cortex) for standard mice (**E**) and mice reared in an enriched environment (**F**). Colour bars: proportion of experiments. Cortical area activated (**G**), size of V1 (**H**) and size of lateral extrastriate cortex (**I**) for both groups. (**G**) p = 0.0074 (one-tailed Wilcoxon Rank-sum test). **p < 0.01. (**H**) p = 0.0164 (one-tailed Welch’s t-test). *p < 0.025. (**I**) p = 0.1669 (one-tailed Welch’s t-test). In (**G**), (**H**) and (**I**), individual data points represent individual animals. Pink triangle: ST female (5 mice in total); blue triangle: ST male (7 mice in total); pink circle: EE female (8 mice in total); blue circle: EE male (8 mice in total). Black line represents the average.

To assess potential organizational and functional changes in the visual cortex, we obtained retinotopic maps with optical imaging of intrinsic signals of adult mice that developed in either an enriched environment (EE) or standard (ST) conditions^40,45^. We hypothesized that the primary visual area of the cortex would be mainly affected as it is more directly susceptible to changes due to the environment. In order to determine this, we first delineated V1 and the lateral extrastriate areas, namely the anterolateral area, the laterointermediate area, the laterolateral anterior area, the lateromedial area and the rostrolateral area, as these were more consistently activated in our cohorts^39,42^. The visual cortical area was larger and more readily identifiable in mice reared in an EE throughout development into adulthood compared to age-matched standard control animals (Fig. 1A–F). Our population of interest included 12 ST mice and 16 EE mice, since these animals reached our inclusion criteria (see “Materials and methods”). This represented 60% of the ST mice imaged, and 76% of the EE mice imaged. The average size of our delimitations was of 3.38 ± 0.54 mm^2^ for ST mice (median of 3.34 mm^2^) compared to 3.86 ± 0.39 mm^2^ for EE mice (median of 4.01 mm^2^; p = 0.0074; Fig. 1G). The differences were mainly explained by a significant increase in size in V1, with an average area of 2.36 ± 0.46 mm^2^ for ST mice (median of 2.42 mm^2^) compared to 2.72 mm^2^ ± 0.34 mm^2^ for EE mice (median of 2.72 mm^2^; p = 0.0164; Fig. 1H,I). The lateral extrastriate area measured in average 1.02 ± 0.28 mm^2^ for ST mice (median of 1.07 mm^2^) compared to 1.14 ± 0.36 mm^2^ for EE mice (median of 1.18 mm^2^; p = 0.1669). Our cohorts were composed of 5 ST females and 7 ST males, and 8 EE females and 8 EE males. No differences based on gender were observed (Table 1). In addition, we observed no particular clustering of data due to litters (Supplementary Fig. S2 and Table S2). No statistics could be performed to establish whether there was a litter effect given the small number of animals per group.

**Table 1:** Gender specific values for cortical area activated through retinotopic mapping show no difference.

Characteristic	Group	p-value	Gender	Average (mm^2^)	St deviation (mm^2^)
Cortical area activated	ST	0.7394	F	3.45	0.52
			M	3.34	0.59
	EE	0.5826	F	3.92	0.37
			M	3.80	0.43
V1	ST	0.6691	F	2.43	0.51
			M	2.31	0.45
	EE	0.9549	F	2.71	0.32
			M	2.72	0.38
Lateral extrastriate area	ST	0.9286	F	1.01	0.30
			M	1.03	0.28
	EE	0.5107	F	1.20	0.27
			M	1.08	0.44

To evaluate if the difference in visual topography relates to visual function, we analyzed different parameters within V1 (amplitude of the response, visual field coverage, scatter index, cortical magnification factor and eccentricity).

### Signal amplitude

**Figure 2:**
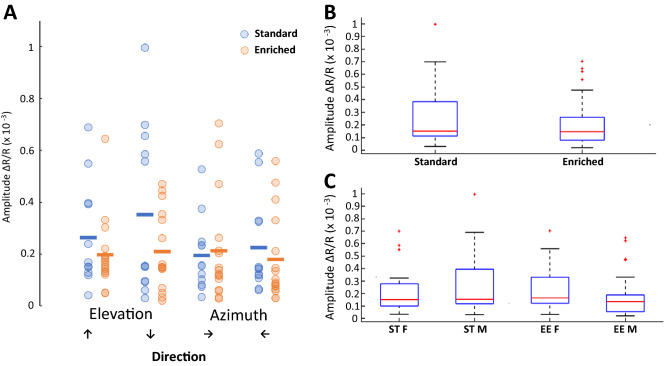
Amplitude in V1. (**A**) Amplitudes as ΔR/R per direction: Up/Down: p = 0.3296; Down/Up: p = 0.1591; Left/Right: p = 0.3466; Right/Left: p = 0.1377 (two-tailed Wilcoxon Rank-sum test). Individual data points represent individual animals. Blue circle: ST mouse (12 mice in total); orange circle: EE mouse (16 in total). Blue line: ST average; orange line: EE average. (**B**) Box plots of overall amplitudes per group (data from **A**): p = 0.0985. N = 12 for ST mice and n = 16 for EE mice. (**C**) Box plot of amplitudes per gender in all directions: ST females (n = 5) vs. ST males (n = 7), p = 0.4579; EE females (n = 8) vs. EE males (n = 8), p = 0.0301.

In order to assess potential differences in signal response, we evaluated the ∆ reflectance/reflectance in our mouse populations. We first established that there were no differences due to the order of presentation of the visual stimuli. Figure 2 shows all averaged signal responses recorded per direction of the stimulation per animal. Descriptive statistics indicated that ST mice had more variability in their response ranges, as their interquartile range was larger (Table 2). No differences observed were statistically significant (direction Up/Down: p = 0.3296; Down/Up: p = 0.1591; Left/Right: p = 0.3466; Right/Left: p = 0.1377; Fig. 2A). However, we noted an overall trend towards higher amplitude levels for ST mice, especially in elevation (Fig. 2A,B). This trend became more apparent when we compared signal responses by gender, with EE male mice having the lowest values and close to significance compared to EE females (p = 0.0301, with p < 0.0250 for significance due to multiple testing; Fig. 2C). There were no differences between ST males and females (p = 0.4579).

**Table 2:** Normalized population ranges for standard and mice that lived in an enriched environment.

Characteristic	Normalized IQR by average		Normalized IQR by median	
	ST (%)	EE (%)	ST (%)	EE (%)
Cortical area activated	26.17	17.36	26.50	16.71
V1	25.44	16.46	24.79	16.48
Lateral extrastriate area	45.44	41.43	43.66	40.08

Amplitude-HB	94.33	34.15	155.76	41.13
Amplitude-BH	143.91	101.53	332.06	142.03
Amplitude-GD	71.96	61.42	90.13	100.85
Amplitude-DG	93.06	93.28	146.14	174.34

Visual field coverage	42.80	40.61	41.20	39.47

Scatter index in azimuth	58.93	59.03	56.05	61.09
Scatter index in elevation	66.98	106.85	72.31	110.80

Magnification factor-average	37.17	49.46	38.80	55.80
Magnification factor-max	39.59	25.98	37.66	25.18
Magnification factor-min	80.96	51.83	81.85	51.39
Magnification factor-range	52.19	18.36	50.07	20.07

Magnification factor-Eccen10	53.71	48.49	57.90	57.03
Magnification factor-Eccen20	27.91	34.40	26.86	42.69
Magnification factor-Eccen30	89.01	34.74	92.21	44.34
Magnification factor-Eccen40	50.24	44.00	53.60	55.64
Magnification factor-Eccen50	47.42	31.53	45.06	35.96
Magnification factor-Eccen60	56.91	33.07	64.19	32.04

Area (# pixels)-Eccen10	54.19	57.57	55.81	60.23
Area (# pixels)-Eccen20	22.07	22.07	21.45	22.01
Area (# pixels)-Eccen30	48.25	25.82	49.11	27.54
Area (# pixels)-Eccen40	59.61	24.45	60.68	24.96
Area (# pixels)-Eccen50	112.46	50.92	149.89	49.85
Area (# pixels)-Eccen60	39.51	34.38	53.51	30.46

### Visual field coverage

**Figure 3:**
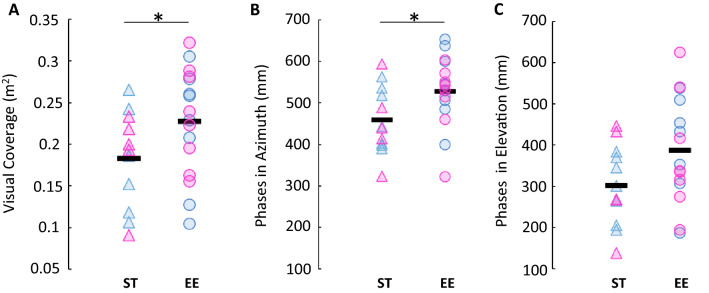
Mice reared in an enriched environment had a greater span of visual field coverage. (**A**) Total visual field coverage by group, p = 0.0311 (one-tailed Welch’s t-test). *p < 0.05. (**B**) Visual span in azimuth by group, p = 0.0193 (one-tailed Welch’s t-test). *p < 0.025. (**C**) Visual span in elevation by group, p = 0.0292 (one-tailed Welch’s t-test). Individual data points represent individual animals. Pink triangle: ST female (n = 5); blue triangle: ST male (n = 7); pink circle: EE female (n = 8); blue circle: EE male (n = 8). Black line represents the average.

As already suspected from the retinotopic maps, the size of visual field coverage in V1 of mice that had grown in an EE was vaster than that of ST mice. The former could see an area of 227 293 ± 64 502 mm^2^, compared to 182 864 ± 55 703 mm^2^ for the latter (p = 0.0311; Fig. 3A). Moreover, the range of phases in both orientations was also greater in EE mice (Fig. 3B,C). These mice could see 528.39 ± 84.77 mm in azimuth and 387.70 ± 129.04 mm in elevation, whereas ST mice could detect the stimulus in 459.45 ± 80.96 mm in azimuth (p = 0.0193, significantly different) and 301.51 ± 96.56 mm in elevation (p = 0.0292, not significantly different). These data suggest that the greater impact was on the range of vision in the horizontal axis.

### Scatter Index

**Figure 4:**
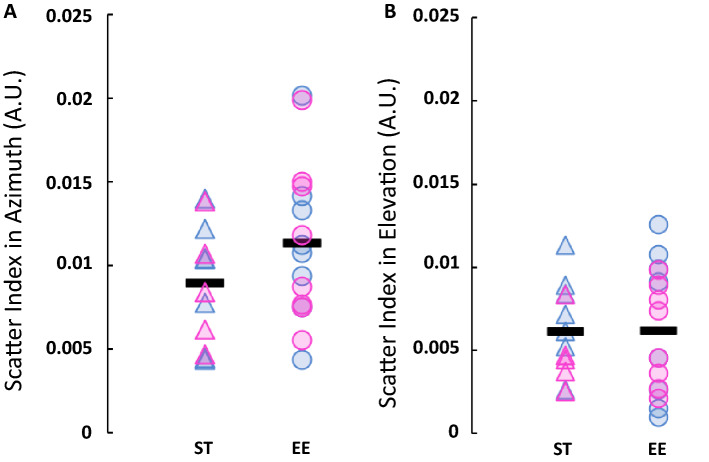
Scatter index in standard mice and mice reared in an enriched environment in V1. (**A**) Scatter index in azimuth in both populations, p = 0.0662 (one-tailed Welch’s t-test). (**B**) Scatter index in azimuth in both populations, p = 0.4788 (one-tailed Welch’s t-test). Individual data points represent individual animals. Pink triangle: ST female (n = 5); blue triangle: ST male (n = 7); pink circle: EE female (n = 8); blue circle: EE male (n = 8). Black line represents the average.

Cang and colleagues^49^, and previous studies from our laboratory have compared the scatter index of different mouse populations^46,50^. This index allows exploring the finer organization of the visual field progression of retinotopic mapping through V1. A low scatter index indicates a higher quality of the retinotopy. Tighter cortical organization can be due to the cortical refinement and is somewhat detectable through optical imaging. Here, we evaluated the local standard deviation from neighboring pixels (sliding windows) along each axis. EE mice had a similar scatter index (even tended to be slightly higher) than ST mice in azimuth [Fig. 4A, 0.0113 ± 0.0037 arbitrary units (A.U.) versus 0.0089 ± 0.0138 A.U., p = 0.0662]. The scatter index in elevation was 0.0061 ± 0.0027 A.U. for ST mice compared to 0.0062 ± 0.0038 A.U. for EE mice (p = 0.4788; Fig. 4B).

### Cortical magnification factor

**Figure 5:**
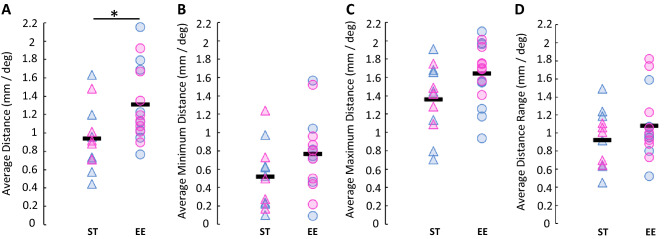
Cortical magnification in standard mice and mice reared in an enriched environment in V1. (**A**) Average distance covered by neighboring pixels in both groups, p = 0.0080 (one-tailed Welch’s t-test). *p < 0.0125. (**B**) Average minimum distance covered by neighboring pixels in both groups, p = 0.0452 (one-tailed Welch’s t-test). (**C**) Average maximum distance covered by neighboring pixels in both groups, p = 0.0253 (one-tailed Welch’s t-test). (**D**) Average distance range covered by neighboring pixels in both groups, p = 0.1106 (one-tailed Welch’s t-test). Individual data points represent individual animals. Pink triangle: ST female (n = 5); blue triangle: ST male (n = 7); pink circle: EE female (n = 8); blue circle: EE male (n = 8). Black line represents the average.

We established the cortical magnification factor (CMF) of each animal by measuring the average distance of the visual field position from each pixel and its immediate surrounding pixels (8 pixels). We expected to see an increase in the average distance covered per set of pixels in the EE mouse population, since their visual field coverage was wider. However, we hypothesized that these differences could be masked by a more refined central vision. Taking these aspects into consideration, we also averaged the minimum and maximum distances, and the range of distances (Fig. 5A–D). As such, the average distance covered by adjacent pixels was indeed larger in mice reared in an EE compared to controls (1.31 ± 0.41 mm/° versus 0.94 ± 0.35 mm/°, p = 0.080; Fig. 5A). We found no significant differences between the averaged minimum distances (0.52 ± 0.34 mm/° for ST mice versus 0.77 ± 0.40 mm/° for EE mice, p = 0.0452; Fig. 5B), maximum distances (1.36 ± 0.38 mm/° for ST mice versus 1.64 ± 0.33 mm/° for EE mice, p = 0.0253; Fig. 5C) nor ranges (0.92 ± 0.31 mm/° for ST mice versus 1.08 ± 0.36 mm/° for EE mice, p = 0.1106; Fig. 5D), although we noted an increase in both the averaged minimum and maximum distances for the population of mice that lived in an EE.

### Eccentricity

**Figure 6:**
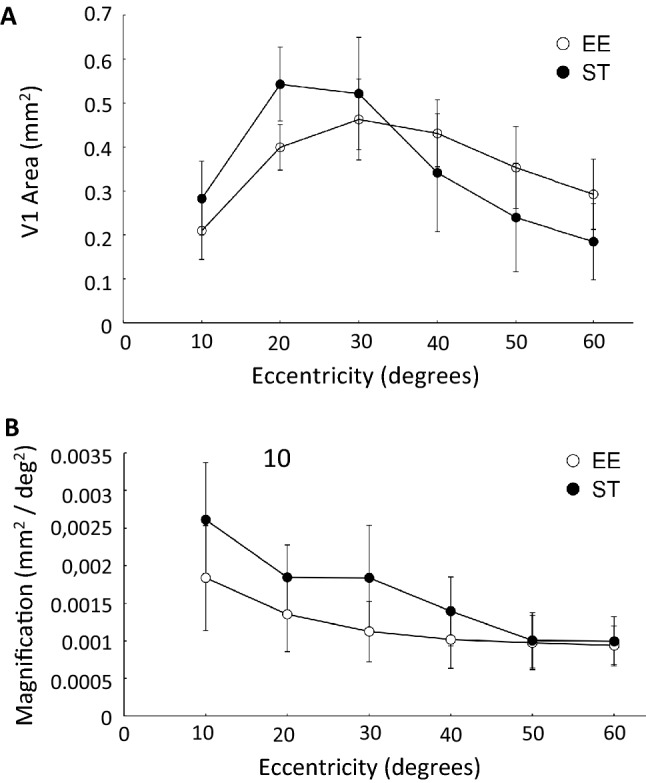
V1 organization per eccentricity of visual field in standard mice and mice reared in an enriched environment. (**A**) V1 area allocated for each range of eccentricity in both populations. There was an effect by eccentricity and by group, when performing repeated measures ANOVA (p < 0.001 for both). (**B**) Cortical magnification for each range of eccentricity in both populations. There was an effect by eccentricity and by group, when performing repeated measures ANOVA (p < 0.001 for the former, p = 0.011 for the latter). Data are presented as average ± SEM. Black filled circle: ST mice (n = 12); white filled circle: EE mice (n = 16).

To further explore the effects of environmental enrichment on cortical magnification, we assessed the area allocated to different eccentricities of the visual field along V1. In order to do so, we compared the area dedicated per range of eccentricity (of 10°) within a comparable area of V1 (Fig. 6A). Interestingly, we found an effect by eccentricity (p < 0.001) and by group (p < 0.001), when performing a repeated measures ANOVA (Levene’s test for equality of variance was first passed). Post hoc analyses revealed a particularly significant difference at 20° (p = 0.0009 from a two-tailed t-test with a threshold level of p < 0.0083), with 0.54 ± 0.08 mm^2^ for ST mice compared to 0.40 ± 0.05 mm^2^ for EE mice. We executed a two-tailed comparison, since we expected a similar distribution of the area per eccentricity between the two groups. In addition, we determined the CMF per eccentricity (Fig. 6B). We performed the same analyses as with area per eccentricity. We also found an effect by eccentricity (p < 0.001) and by group (p = 0.011). Taken together, these data strongly suggest that enrichment during development has profound effects on cortical organization and function geared towards a better integration of the surroundings; more specifically, an increased size of V1, a greater visual field and a refined visual cortex organization.

### Gender

Except for the tendency reported above within the EE population regarding amplitude, no differences or trends based on gender were observed in all the other parameters (Figs. 1, 2, 3, 4, 5).

## Discussion

In this study, we report that an enriched environment (EE) during various critical periods of development, from the prenatal period to adulthood, has an impact on the structure and function of the visual cortex. Specifically, mice reared in an EE developed a larger visual cortical area compared to standard (ST) mice. This effect was mainly due to differences in the size of V1. To understand how this difference translates functionally, we evaluated parameters of vision in V1, of which the visual field coverage, the cortical magnification factor and the eccentricity topography were significantly affected.

In order to assess the differential effects of the two environments, we first delineated V1 and the lateral extrastriate cortex through retinotopic mapping obtained by intrinsic signal optical imaging (ISOI). There were striking increases in the dimensions of V1 in the population of mice reared in an EE. Further analyses demonstrated that these expansions were not based on gender, as both females and males benefited from their EE. We had initially hypothesized that an EE would provide a wider range of effects with subjects falling somewhere on the spectrum of low to high responders of novelty and diversification depending on how much individual mice actively explore and engage with the environment they are in, even if they have genetically identical backgrounds^51,52^. Indeed, various studies, although not all, have indicated that they are higher levels of variability within EE mice, depending on what is measured^52,53^. Contrary to our expectations, the levels of variability appear similar amongst mice from either an ST or EE, with the EE mouse population tending towards narrower interquartile ranges. There are a large number of studies with differing enrichment paradigms that emphasize on particular elements (relevance of the context of the animal’s environment reviewed in^53^). Here, we established a level of enrichment that was minimal (few variables) and easily maintained that showed measurable outcomes to better understand enrichment. For illustrative purposes, we focus on the study by Freund and colleagues^51^. They followed a mouse cohort of 40 inbred female mice, C57BL/6N, from as many litters as possible, that was placed in a rather complex yet static environment at 4 weeks old and remained there for 3 months. They noted that individuality increased with age. Our results do not show this trend; however, our EE mouse population was much smaller (16 subjects). In addition, our small variability could be the result of all mice born from only three sets of litters (two pregnant females per cage, three times) rendering our cohort more uniform (reviewed in^54^). Furthermore, novelty was introduced twice a week in our cages.

Developmental studies in both animal models and humans have demonstrated that the pace of development varies according to different factors (reviewed in^34^). Critical periods are flexible: comfortable and stimulating environments are permissive of longer plasticity windows. Gopnik^55^ argues that there are explore-exploit tensions that allow the transition from childhood to adulthood, where ‘explore’ is a learning phase and ‘exploit’ a phase for skilled action. To avoid missing completed developmental stages, we made our recordings in young adulthood. We also placed our pregnant mice into their respective environments 1 week prior to giving birth, since the perinatal/prenatal period appears to be a sensitive period for cortical surface area development^37^.

From our results, we can therefore cautiously speculate from our EE a possible threshold for beneficial impacts. More specifically, our EE paradigm offers enough stimulation to have a positive influence on brain structure and function as determined by the parameters studied. This doesn’t discard the possibility that greater enrichment could trigger more complex and graded effects. However, this raises the question of what are appropriate levels of enrichment? Calhoun’s studies from the 1960s and 1970s, where he created mouse or rat utopias, clearly indicate that there are ceilings to enrichment paradigms^56^. Too little causes deficiencies and too much causes excesses, both ends of the spectrum inducing anxiety. In line with these discoveries, male Ts65Dn mice with a deletion in chromosome 16, a model of Down Syndrome, did not profit from environmental enrichment; on the contrary, they had decreased learning capacities compared to mice living in a less EE^57^. A previous study from the same group had found that females benefited more than males from an EE with regards to spatial memory assessed by the Morris water maze^58^. Female mice appear more susceptible to stress than males^54^ furthering the notion that optimal living conditions allow for greater adaptability. However, more studies are needed to elucidate whether or not there is gender advantage^59^. We did not observe one with the parameters we measured.

The visual system of our EE mouse population was differently solicited than our ST mouse population; and therefore, the organization of the visual map was functionally affected. Quite interestingly, environmental enrichment during development stimulated the visual system to detect wider horizons. This was observed in both axes, although predominantly in azimuth. One could argue that our enrichment was more prominent in that plane. Interestingly, a recent study has demonstrated that humans have radial asymmetries of the visual field, where visual cortical area dedicated to the azimuth predominates^60^. The latter topography correlates with greater visual task performance in the horizontal axis (better acuity). In addition, visual field attentional redistribution has been shown in humans following training. For instance, the regular practice of sign language causes resources to focus on the inferior visual field as assessed by visual search task^61^. Signers exhibit an improved attention in the lower visual field.

An in-depth analysis of the organization of V1 showed that the two mouse populations have different topographies within V1. Indeed, larger V1 areas were dedicated to smaller eccentricities in the visual cortex of ST mice. From 40° of eccentricity onwards, there was a switch where bigger areas of V1 of mice from an EE are devoted, compared to ST mice. The widest gap between the two groups was particularly evident at 20° of eccentricity, with ST mice exhibiting a marked increase in comparison to EE mice. ST mice also had a pronounced change from the allocation of area to each range of eccentricities from closest to furthest, whereas it was more constant in EE mice. Moreover, there was an effect on the overall cortical magnification factor (CMF), but also per range of eccentricity. Within comparable functional areas of V1 as determined by the maximal eccentricity interval, ST mice elicited bigger CMF, at all eccentricities. However, mice reared in an EE had a wider field of vision and surface of V1, and ultimately a wider average distance covered by sets of neighboring pixels. This data suggests that these discrepancies could originate from a lack of stimulation within the ST mouse population and an increased focus on what is right in front of them, while the visual cortex of the EE mouse population had to accommodate for a diversified reality.

Although it was suspected that only V1 would show drastic effects (as it is the region that is mostly affected by the environment within the visual cortical hierarchy^62^), we noted that differences were apparent throughout the delimited areas between the two populations based on the quality of the retinotopic maps. Perhaps this is due to an important circuitry linking V1 to the extrastriate areas present in mice and more prominent than in other higher mammalian species^63^. Although the size differences were only significant for V1, we argue that this is probably due to the fact that V1 is the largest area and therefore differences are more readily observable. More work into the subtleties of these differences will shed interesting light on the effects within the mouse visual cortex organization. Given the recent exponential growth of research dedicated to the mouse visual system, it is a great model to keep exploring the benefits of environmental enrichment. Especially since enrichment in general is not modality specific (reviewed in^53^).

In conclusion, our study provides clear measurable effects of an EE on the structure and function of the developing visual cortex, as determined by ISOI. Functional delimitations of surface area have seldom been performed in animal or human studies alike, hence these data add to our understanding of how flexible these processes are. Further discerning between all the variables will allow to better implement changes throughout development, certain elements potentially being more relevant at specific ages. Not only the nature but also the amount of enrichment needs to be carefully addressed^33^.

## Supplementary Information

Supplementary Information.

## Data Availability

The datasets generated during and/or analysed during the current study are available from the corresponding author on a reasonable request.
