# Supplementary material for: Enriched environment exposure during development positively impacts the structure and function of the visual cortex in mice
Source: Sci Rep. 2023 Apr 29;13:7020. doi: 10.1038/s41598-023-33951-0 (PMC10148800; doi:10.1038/s41598-023-33951-0)
Supplement: Supplementary file 1 — Supplementary Information. [file 41598_2023_33951_MOESM1_ESM.pptx]

## Slide 1
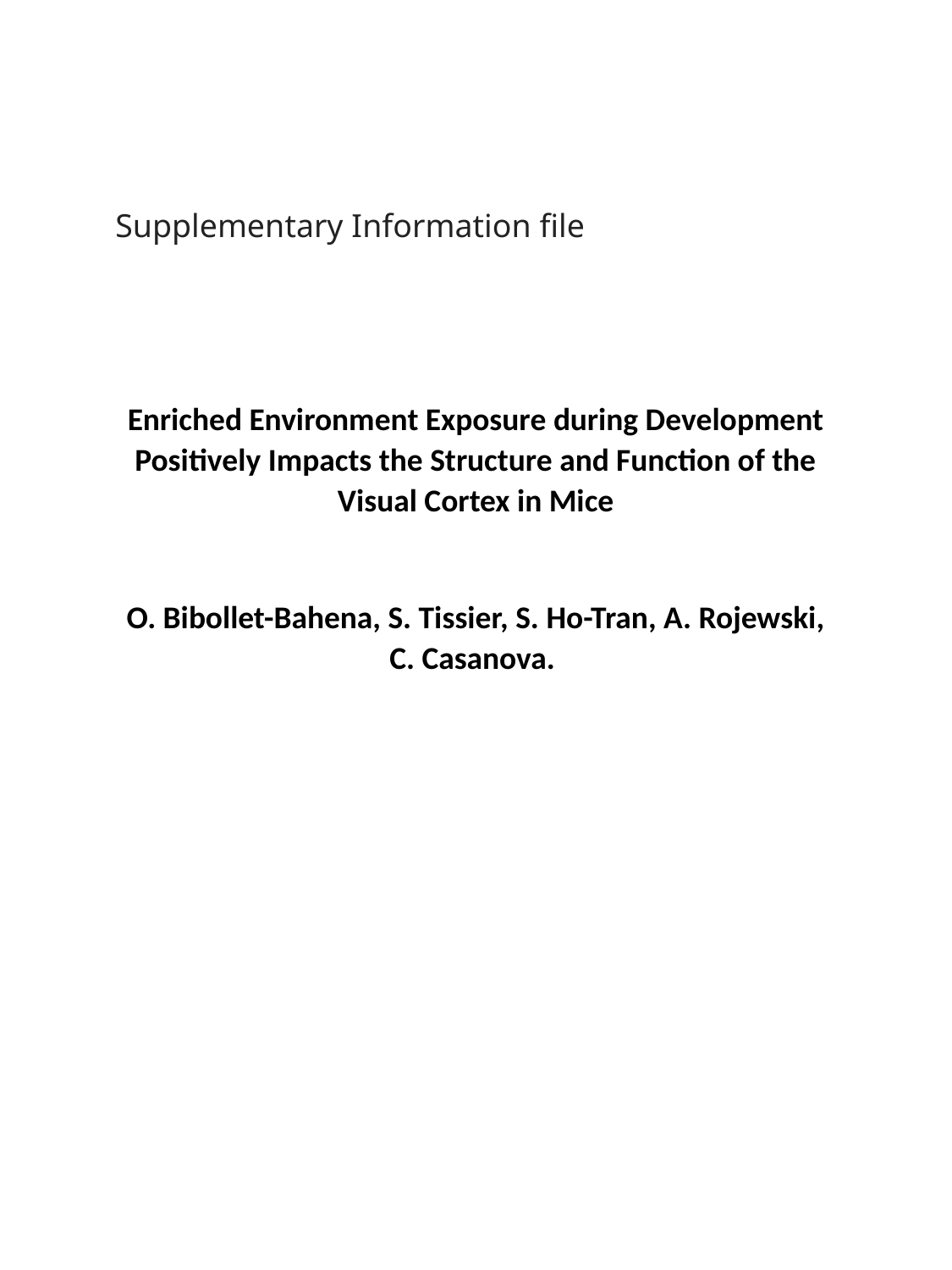

Supplementary Information file
Enriched Environment Exposure during Development Positively Impacts the Structure and Function of the Visual Cortex in Mice
O. Bibollet-Bahena, S. Tissier, S. Ho-Tran, A. Rojewski, C. Casanova.

## Slide 2
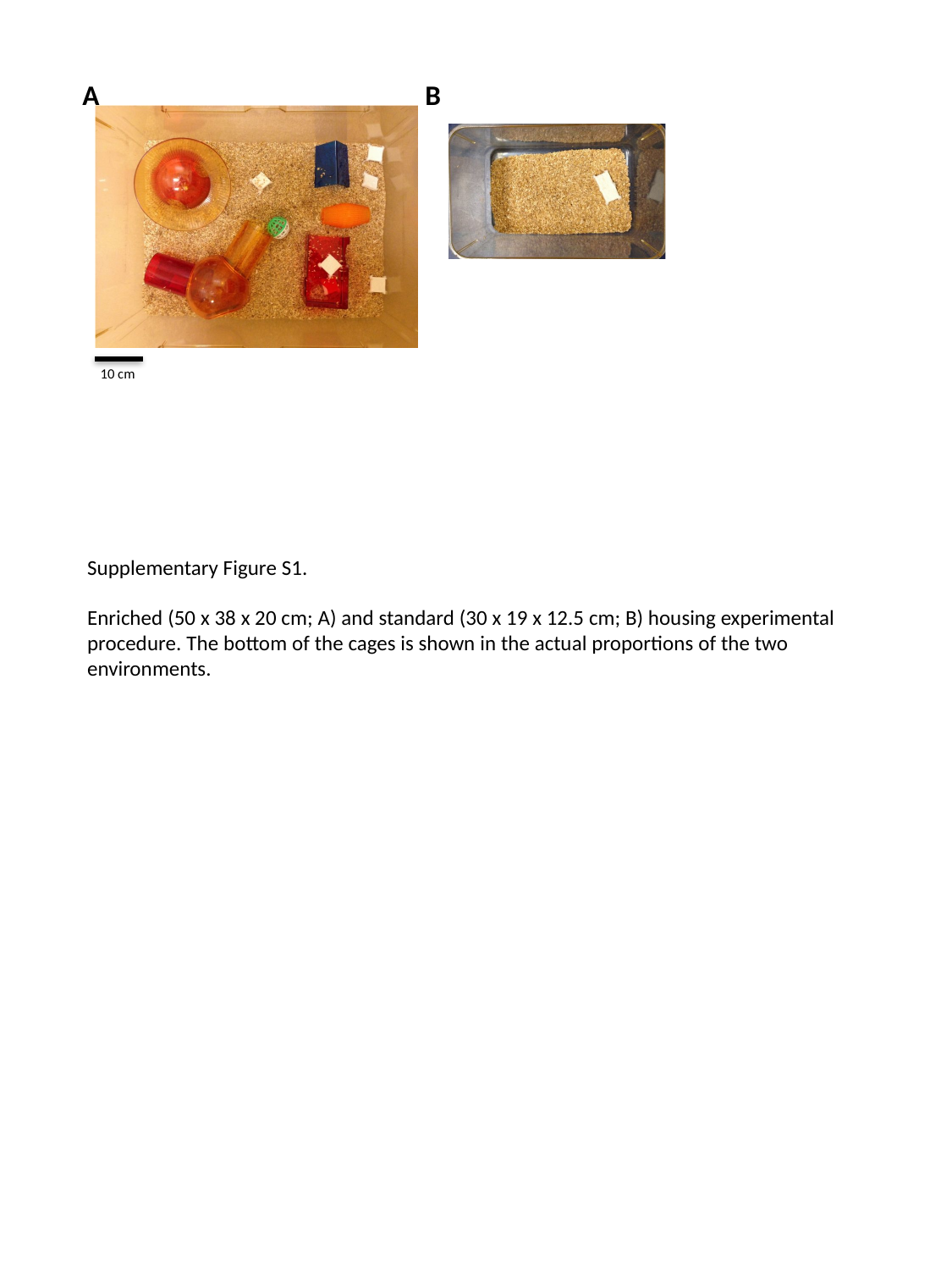

A
B
10 cm
Supplementary Figure S1.
Enriched (50 x 38 x 20 cm; A) and standard (30 x 19 x 12.5 cm; B) housing experimental procedure. The bottom of the cages is shown in the actual proportions of the two environments.

## Slide 3
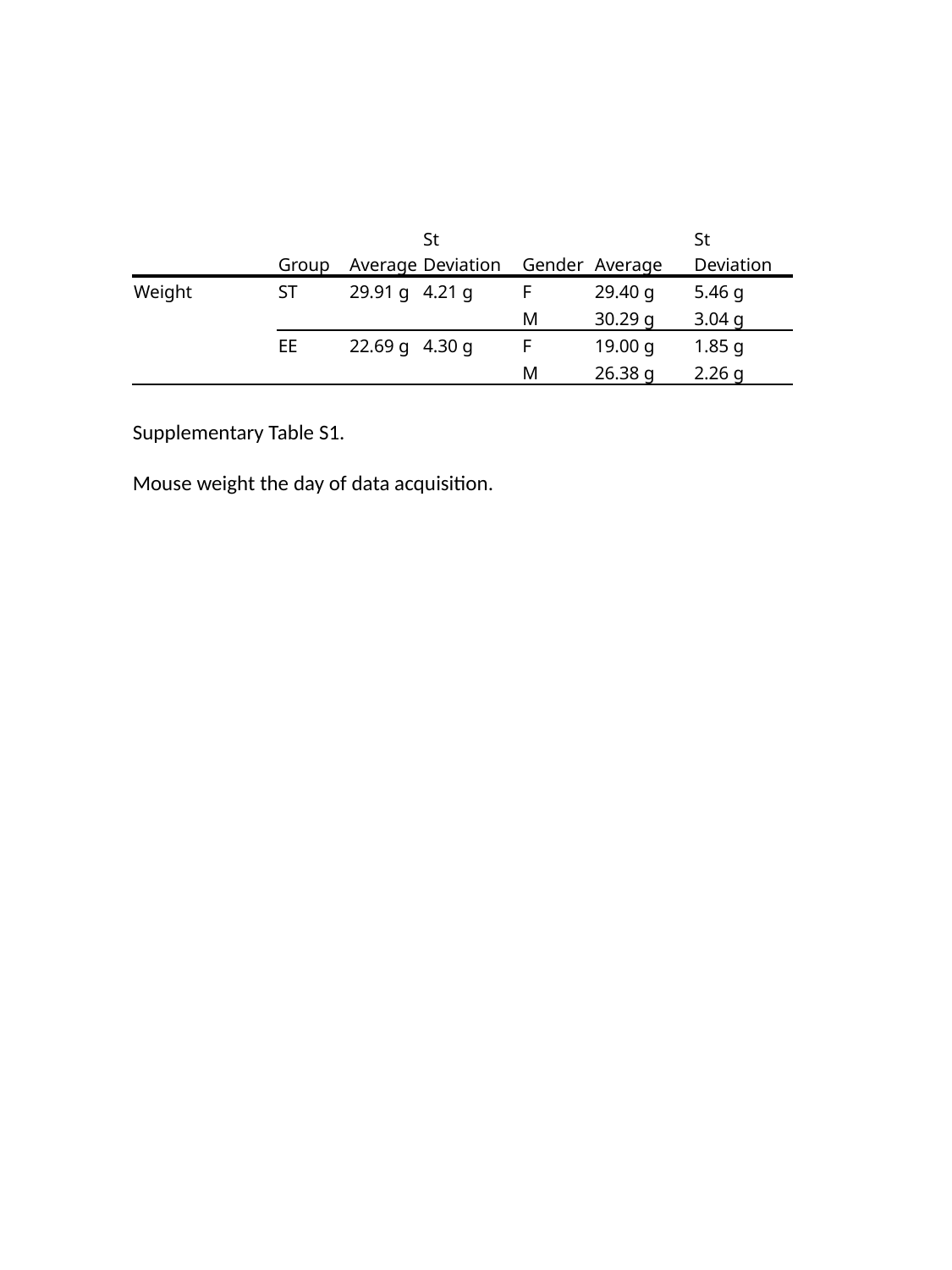

| | Group | Average | St Deviation | Gender | Average | St Deviation |
| --- | --- | --- | --- | --- | --- | --- |
| Weight | ST | 29.91 g | 4.21 g | F | 29.40 g | 5.46 g |
| | | | | M | 30.29 g | 3.04 g |
| | EE | 22.69 g | 4.30 g | F | 19.00 g | 1.85 g |
| | | | | M | 26.38 g | 2.26 g |
Supplementary Table S1.
Mouse weight the day of data acquisition.

## Slide 4
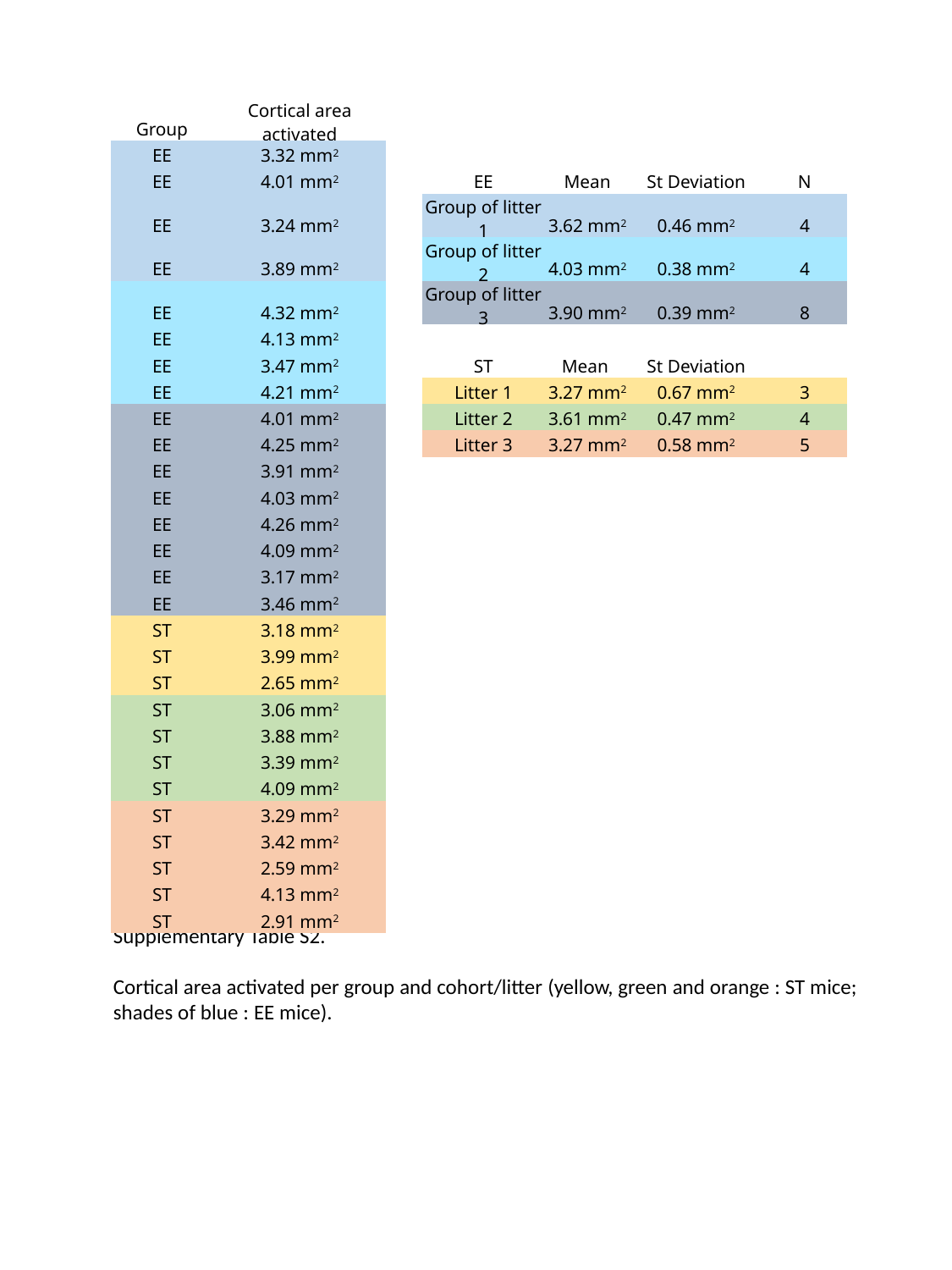

| Group | Cortical area activated | | | | | |
| --- | --- | --- | --- | --- | --- | --- |
| EE | 3.32 mm2 | | | | | |
| EE | 4.01 mm2 | | EE | Mean | St Deviation | N |
| EE | 3.24 mm2 | | Group of litter 1 | 3.62 mm2 | 0.46 mm2 | 4 |
| EE | 3.89 mm2 | | Group of litter 2 | 4.03 mm2 | 0.38 mm2 | 4 |
| EE | 4.32 mm2 | | Group of litter 3 | 3.90 mm2 | 0.39 mm2 | 8 |
| EE | 4.13 mm2 | | | | | |
| EE | 3.47 mm2 | | ST | Mean | St Deviation | |
| EE | 4.21 mm2 | | Litter 1 | 3.27 mm2 | 0.67 mm2 | 3 |
| EE | 4.01 mm2 | | Litter 2 | 3.61 mm2 | 0.47 mm2 | 4 |
| EE | 4.25 mm2 | | Litter 3 | 3.27 mm2 | 0.58 mm2 | 5 |
| EE | 3.91 mm2 | | | | | |
| EE | 4.03 mm2 | | | | | |
| EE | 4.26 mm2 | | | | | |
| EE | 4.09 mm2 | | | | | |
| EE | 3.17 mm2 | | | | | |
| EE | 3.46 mm2 | | | | | |
| ST | 3.18 mm2 | | | | | |
| ST | 3.99 mm2 | | | | | |
| ST | 2.65 mm2 | | | | | |
| ST | 3.06 mm2 | | | | | |
| ST | 3.88 mm2 | | | | | |
| ST | 3.39 mm2 | | | | | |
| ST | 4.09 mm2 | | | | | |
| ST | 3.29 mm2 | | | | | |
| ST | 3.42 mm2 | | | | | |
| ST | 2.59 mm2 | | | | | |
| ST | 4.13 mm2 | | | | | |
| ST | 2.91 mm2 | | | | | |
Supplementary Table S2.
Cortical area activated per group and cohort/litter (yellow, green and orange : ST mice; shades of blue : EE mice).

## Slide 5
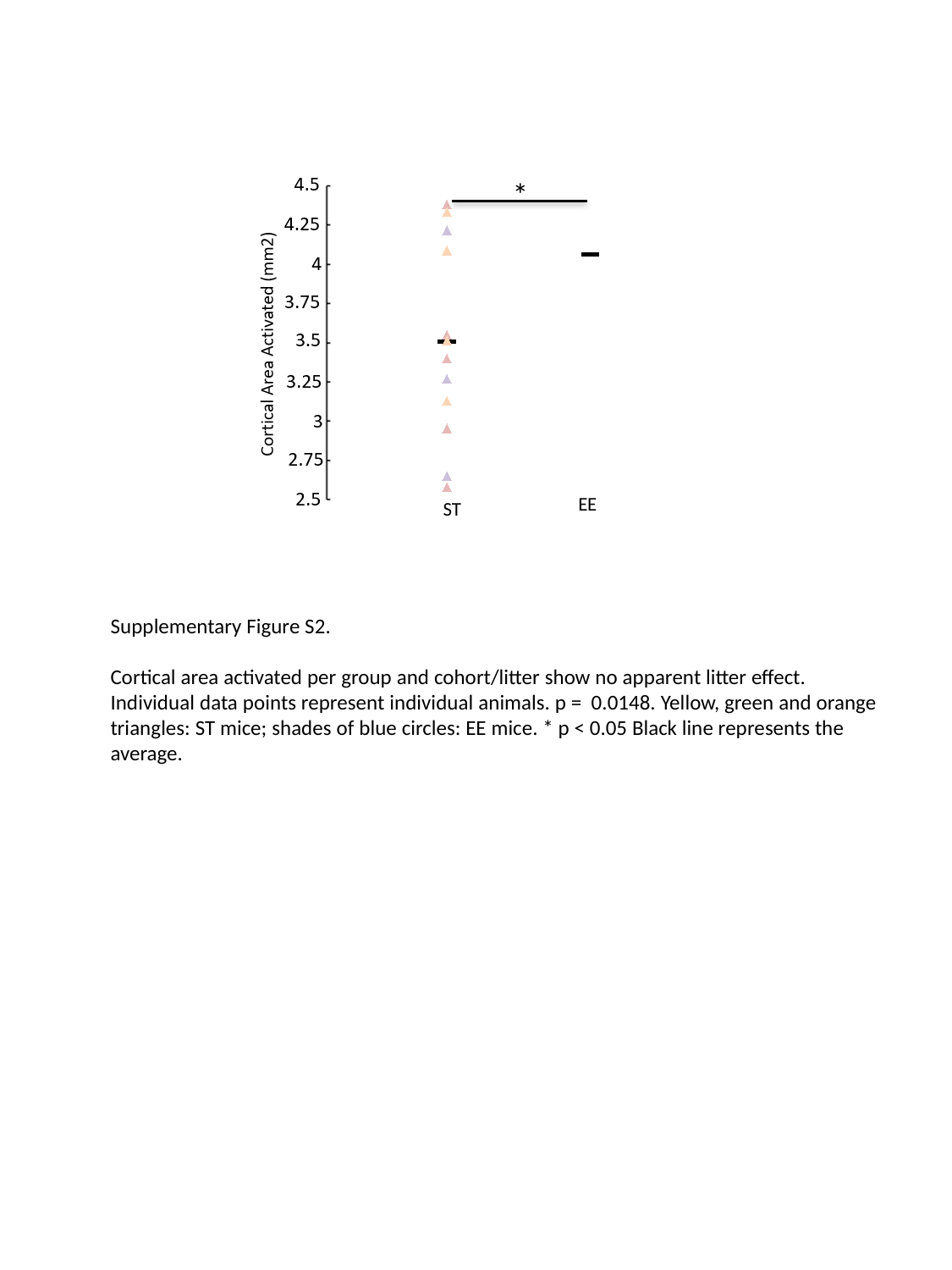

### Chart
| Category | | | | | | | | |
|---|---|---|---|---|---|---|---|---|ST
*
Supplementary Figure S2.
Cortical area activated per group and cohort/litter show no apparent litter effect. Individual data points represent individual animals. p = 0.0148. Yellow, green and orange triangles: ST mice; shades of blue circles: EE mice. * p < 0.05 Black line represents the average.
